# From society to cells and back again: new opportunities for discovery at the biosocial interface

**DOI:** 10.1007/s44155-022-00007-z

**Published:** 2022-03-09

**Authors:** Thomas W. McDade, Kathleen Mullan Harris

**Affiliations:** 1 Department of Anthropology and Institute for Policy Research, Northwestern University, Evanston, IL 60208 USA; 2 Department of Sociology and Carolina Population Center, University of North Carolina at Chapel Hill, Chapel Hill, NC 27516 USA

**Keywords:** Biosocial, Health disparities, Life course, Human development, Interdisciplinary

## Abstract

A new generation of community- and population-based research is combining measures of social context, experience, and behavior with direct measures of physiology, gene sequence and function, and health. Studies drawing on models and methods from the social and biological sciences have the potential to illuminate the multilevel mechanisms through which experience becomes biology, and to move past decontextualized and reductionistic approaches to human development, behavior, and health. In this perspective we highlight challenges and opportunities at the biosocial interface, and briefly discuss COVID-19 as a case study demonstrating the importance of linking across levels of analysis.

## Introduction

For more than 100 years, social and behavioral scientists have aimed to unravel the complex interactions among genes and environments that shape human behavior, biology, and health. We have entered a unique moment, with unprecedented opportunities for discovery resulting from the integration of physiologic and genomic data into community- and population-based studies that contain in-depth measures of social context and experience across the life course. In this perspective, we highlight challenges and opportunities emerging at the interface of the social and biological sciences, and underscore how work in this area can fundamentally change how we think about human biology, behavior, and health.

## The challenge

In 2000, we celebrated the sequencing of the human genome as a tremendous scientific achievement and triumph for humanity. Francis Collins, who led the Human Genome Project and now directs the National Institutes of Health, declared “... we have caught the first glimpse of our own instruction book, previously known only to God.” The UK Science Minister Lord Sainsbury proclaimed “We now have the possibility of achieving all we ever hoped for in medicine” [1]. With expectations set so high, disappointment was sure to follow, and less than 10 years later hyperbole gave way to head scratching as the genetic origins for common traits and diseases were nowhere to be found [2].

While the search for the “missing heritability” continues, an overlooked contribution of the Human Genome Project is that we can now answer this question: How many genes are in the human genome? When the project started in 1990, educated guesses hovered around 100,000. Today, it is approximately 20,000. That is about the same number of genes as the roundworm, and fewer than the grape and tomato [3].

A downsized human genome, plus modest success in “achieving all we ever hoped for in medicine,” challenge us to move past an exclusive focus on the genetic origins of complex phenotypes, and instead consider how genes and environments iterate over the course of development. We are also challenged to define a biosocial approach that pushes beyond the reductionist explanatory frameworks common in biomedicine as well as assumptions of biological determinism widely held among the general public. Fortunately, the pieces are now in place to do just that.

## The opportunity

Social and behavioral scientists routinely design studies that include rich measures of the environment. That is, where do people live and with whom do they interact? What challenges and opportunities do they face as they inhabit various roles and positions over the life course, navigating a wide range of social institutions and hierarchies? An important contribution here is that many of these studies are community-based and population-representative, and include large numbers of participants, as well as measurement at multiple levels (e.g., macro-structural, community, interpersonal, individual) and time points. This approach has generated countless insights into how structure and agency define the human experience, and it has emerged as a powerful framework for illuminating the social determinants of health [4].

Historically, social scientists have relied on administrative records (e.g., vital statistics) or self-reported, survey-based measures of health (e.g., participant ratings of general health, and/or more detailed reports of symptoms, diagnoses, and functional limitations). Insight into biological mechanisms is thereby limited, in contrast with biomedical studies that can dig deeper by collecting data in controlled clinic- or lab-based settings to assess the structure and function of cells, tissues, and organ systems. These studies, however, often rely on smaller numbers of participants who live close to academic medical centers and who are recruited based on pre-existing criteria related to a condition of interest. Generalizability is limited, and social/behavioral factors are rarely considered beyond simple measures of socioeconomic status or health-related behaviors like smoking.

Recent technological advances now allow us to bridge the gap between field- and clinic-based approaches to understanding human biology, development, and health. For example, blood samples are routinely assayed for biomarkers of physiological function and health, but require substantial infrastructure to facilitate collection (venipuncture), processing (centrifugation and pipetting aliquots of plasma), and transport (a cold chain). By contrast, low cost, “field friendly” alternatives to venipuncture can, in many cases, provide access to the same information in a small vial of saliva or urine, or a couple of drops of blood collected from a simple finger stick [5, 6]. Self-sampling of the human microbiome—which is emerging as an important link between ecology and health—is low cost and feasible for large-scale applications [7]. Assay technologies now allow the quantification of proteins, gene transcripts, epigenetic marks, and DNA sequences in smaller quantities of sample, at higher resolution, at lower per sample costs [8–10]. Wearable technologies, portable monitors, and smart phone apps are all expanding the options for measuring sleep, physical function and activity, blood pressure and cardiovascular function, and aspects of brain development and activity [11–14]. Many of these methods (e.g., finger stick dried blood spot sampling) have been implemented in low-resource settings globally [6]; others may pose logistical or financial constraints that currently limit application to higher income countries. In short, it is now possible to collect biological data on large numbers of people in naturalistic settings—representing a wide range of race/ethnic, gender, socioeconomic, and geographic groups—at relatively low cost and participant burden.

Consider the possibilities here. For the first time we are at a point where we can integrate in-depth measures of contexts, experiences, and behaviors with direct, objective measures of physiology, gene sequence and function, and health. We have arrived at a moment when we can achieve this multilevel integration in large, diverse, representative samples, with multiple measures over time. Combine these emerging “big data” resources with computational advances in dealing with high dimensional datasets, and there is an unprecedented opportunity for discovery at the biosocial interface.

## Putting the “bio” in “biosocial”

Historically, social scientists have positioned themselves outside the body, only occasionally considering how social contexts and experiences engage biological processes to shape developmental and health outcomes. But with new methodological tools, scholars in anthropology, demography, economics, psychology, and sociology are finding common ground with colleagues in epidemiology and public health, medicine, and genomics [15–18].

There are several reasons why this is a promising development. First, biological measures reveal the mechanisms through which socioeconomic, spatial, and psychosocial contexts “get under the skin” to shape developmental trajectories and health outcomes. They provide more direct and objective measures of health status than self-reports, and they can be used to identify which experiences and environments are most toxic. In other words, biological measures offer access to embodied information that helps illuminate the impact of experience on health, even if this information is below the threshold of perception or cannot be articulated by participants.

Second, biological measures provide direct information on pathophysiological processes before the emergence of clinical disease. For example, the relative levels of risk markers for cardiovascular disease (e.g., blood pressure, lipids, C-reactive protein) remain relatively stable, or “track” from childhood into adulthood [19]. Furthermore, young adults in their 20 s and 30 s, thought to be in the healthiest years, rarely seek medical care even though asymptomatic conditions like hypertension, high cholesterol, and glucose intolerance lay the foundation for future morbidity and mortality [20, 21]. Using biological measures to tap into pre-disease pathways can therefore identify groups of individuals at-risk prior to the development of disease, as well as the processes that may exacerbate or ameliorate this risk. These measures also help identify which physiological pathways (e.g., aspects of neuroendocrine, cardiovascular, metabolic, and/or immune function) are most involved in linking the contexts and health outcomes of interest.

Third, biological measures can be used as tools for policy evaluation. While clinical care is critical to improving health outcomes for individual patients, social policy interventions have the potential to impact large numbers of people by addressing upstream contexts and experiences that contribute to—or ameliorate—social inequalities in a wide range of developmental and health outcomes. An important challenge here is evaluating impact. For instance, a recent analysis documents a 30 year life expectancy gap between residents of the Gold Coast (90 years) and Englewood (60 years) neighborhoods of Chicago, located only nine miles apart [22]. Calls for action have followed, and it would be very useful to know which policies are most effective in reducing this inequity before another two or three generations of Englewood residents have to come of age with such a stark mortality disadvantage. Biological measures can provide near term information (weeks to months following an intervention) that forecasts likely long term (years to decades) impact on health. In other words, if we collect biological measures before and after the implementation of a social policy, we do not have to wait for people to die to determine if it is likely to be effective in reducing the mortality gap.

Furthermore, biological measures can generate insight into the health impacts of programs not necessarily designed to affect health. Social scientists often focus on policies to improve human capital outcomes like educational attainment or workforce participation, but ignore potential for spillover effects in other domains like health. The Moving to Opportunity (MTO) demonstration project provides a case in point. Originally designed to investigate the impact of residential contexts on educational attainments and income, families in public housing were randomly selected to receive a voucher that subsidized their move into a low poverty neighborhood. Ten to fifteen years later, the intervention had limited effects on education and income, but large impacts on health: assignment to the low poverty group resulted in a 13–19% reduction in obesity and 22% reduction in diabetes [23]. Given the high costs of health care, biological measures may add an important, but often overlooked, component to cost/benefit analyses of specific social policies and programs.

Lastly, the integration of genomic measures into social science surveys allows us to challenge the common understanding of genes as deterministic forces fixed at birth. As underscored by the case of the missing heritability, we gain relatively little from information on gene sequence alone while analyses of gene-environment interplay enhance explanatory power: the role of genes in smoking behavior is lower in states with higher cigarette taxes; neurotransmitter polymorphisms moderate the impact of childhood maltreatment on risk for depression; and genetic similarity among adolescent friends is increased in schools with greater levels of social inequality [24–26]. In addition, polygenic scores show promise in overcoming limitations associated with candidate gene and genome-wide studies, and provide new opportunities for investigating how “genetic inheritance” may differentially influence outcomes across social contexts and stages of the life course [18]. Furthermore, analyses of gene expression (the transcriptome) and biochemical modifications to DNA and its packaging (the epigenome) illuminate intra-cellular mechanisms through which the body “remembers” prior experiences and contexts, and they document the relevance of forces “outside the body” to multiple levels of gene function. For example, social isolation increases the expression of pro-inflammatory genes, lower socioeconomic status reduces expression of glucocorticoid response elements, and family adversity predicts levels of DNA methylation in genes associated with immune function [27–29].

The availability of genomic data is allowing social scientists to advance longstanding interests in the dynamic interactions among genes and environments over the life course. For scholars working outside this area, genetic data can be used to control for genetic influences on social, behavioral, and health outcomes of interest in order to foreground the causal impacts of environmental factors [30]. Most importantly, we now have an opportunity to advance our understanding of the genome as a dynamic substrate that incorporates information from the environment over developmental time, and to underscore the critical point that we cannot understand gene regulation and function without reference to experience and context.

## The importance of socializing biology

While social scientists are increasingly embracing biological data to make important contributions in each of these areas, we propose an additional, and perhaps more ambitious agenda: to reframe how we conceptualize and study human biology. For the most part, research in the biological sciences is an exercise in reduction that seeks to illuminate the cellular and molecular mechanisms that drive processes—and diseases—of interest. Explanations for disease are located “inside the body,” with little, if any attention given to a causal role for social or other contextual factors. Consider the hyperbole following the sequencing of the human genome, and the seemingly endless stream of media reports on the “gene for (insert favorite trait here)” as evidence for how willing we are to privilege genes as important determinants of our behavior, development, and health. Clinical medicine is also, for the most part, trapped inside the body as it seeks to isolate single, proximate factors as causes of disease and targets for treatment [31]. Blockages in coronary arteries cause heart attacks. Viruses cause infections. Tumors cause cancer.

But social isolation also causes heart attacks, lower socioeconomic status increases susceptibility to viral infections, and psychosocial stressors promote tumor growth [32–34]. In fact, a strong case can be made that social relationships and social rank are the two most important determinants of health [4, 35, 36]. Humans are long-lived, social creatures who depend on one another for survival, and it should therefore come as no surprise that access to resources, and the quality and quantity of connections with other people, are key determinants of how long we live. It should also come as no surprise that we can detect physiological signatures of social experience and context, and that we can use biological measures to generate novel insights into how experiences of poverty and social isolation translate into poor health and early death [20, 21].

In short, human biology is a social biology. It is therefore essential that we “socialize” our conceptual models and study designs accordingly. How do we do this? First, we draw on theoretical frameworks and analytic techniques that integrate across levels of analysis and locate causation at multiple levels, from the distal upstream to the more proximate downstream factors inside and outside the body [14]. Yes, viruses cause infections. But there are reasons, outside the body, why some people are more likely to be exposed to viruses. Yes, tumors cause cancer, but exposures outside the body influence patterns of gene expression in white blood cells that target malignancy. We use the term “social” very broadly: a wide range of contexts and experiences may be relevant here, including socioeconomic status (which has been conceptualized as income, education, occupation, wealth, and/or subjective rank), quality and quantity of interpersonal relationships, aspects of the built environment, structural racism, cultural norms and expectations, exposure to adversity or trauma, and more. [14, 37, 38].

Second, we conduct research in diverse, community-based settings in order to represent, and evaluate, the wide range of experiences and exposures that contribute to variation in human biology, development, and health over the life course. Third, we move past the common assumption that human biology can only be studied in the lab or clinic. By taking our methods into the community, where everyday people are living their everyday lives, we encourage an epistemological reframing of human biology as complexly determined by multiple forces, inside and outside the body.

It is important to emphasize that this is not a repudiation of reductionism. Studies of molecular mechanisms advance our understanding of disease, as do analyses of the social and structural determinants of health. We argue here for a complementary approach that transcends the dichotomy between proximate and distal levels of analysis, that conceptualizes the biological and the social as interdependent and mutually constituting forces, and that links across phenomena inside the body and outside the body.

## A socialized biology, not sociobiology

For some social scientists, using the words “social” and “biological” in the same sentence serves as a discomfiting reminder of a deep history of typological and essentialist thinking that has employed biological measures as tools of marginalization and oppression, and a recent history of biomedical abuses in engaging with communities of color [39, 40]. Such misuses and abuses are not inevitable, however, and there are costs to throwing out the baby with the bath water. For example, if we assume that all attempts to integrate the biological and social are destined to perpetuate simplistic notions of biological determinism, then we have to build a firewall between the two. This is the path of least resistance, and it largely reflects—and reinforces—current trends toward disciplinary and subdisciplinary specialization and siloing. Going further, isolating the biological from the social promotes an outdated Cartesian dualism that denies the corporeality of the human experience, and it turns its back on insights from modern biology regarding developmental plasticity and the sensitivity of the human body to the environments in which it inhabits [41, 42].

It also perpetuates among the general public a status quo (mis)understanding of biology as static and immutable, with genes as primary drivers of developmental, behavioral, and health outcomes. In other words, commonly held folk models of causation do not recognize the contingency of biological processes, the complexity of interplay between genes and environments over the life course, nor the extent to which biological systems depend on input from the environment to guide their development and function [43]. What better way to confront simplistic thinking about biology than to engage in research that undermines the fallacious foundation of genetic determinism? What better way to undermine the phrase “gene for (insert favorite trait here)” than by showcasing how social contexts actually shape gene structure, activity, and relevance to outcomes we care about?

And lastly, in isolating the biological from the social we deny ourselves the opportunity to shed light on how the unequal distribution of resources and opportunities becomes embodied and perpetuates social inequities in health. For example, rates of hypertension and pre-term delivery are consistently higher for Black adults in comparison with white adults in the US. Why is this the case? Attempts to account for these differences with relatively crude measures of socioeconomic status typically fail, leading some in the biomedical community to claim that there must be a genetic explanation [44, 45]. Are we comfortable ceding control of this narrative, which locates the origins of these inequities exclusively inside the body and, more perniciously, constructs people who identify as Black or white as genetically distinct racial groups?

We can, and should, critique typological and essentialist thinking in biomedicine and society at large, as many prominent scholars have already done [39, 40, 46]. But we can also do more. We can analyze genetic data to demonstrate how genes do not map onto socially defined racial and ethnic groups, and we can make the point that relatively little variance is explained by studies focusing exclusively on genetic contributions to complex traits. We can use epigenetic data to document how poverty sculpts wide swaths of the genome [29]. We can use physiologic data to reveal the hidden toll that discrimination takes on the bodies of individuals who may be marginalized based on the color of their skin [47, 48]. If we, as social scientists, feel empowered to apply concepts and methods from the biological sciences then we can provide more compelling evidence attesting to the importance of social and contextual factors, and play a more active role in shaping scientific and public understandings of the causes of inequalities in developmental and health outcomes. Indeed, there has been a slow but upward trend in biosocial research publications in social science journals, especially when the integration of biological theory and data serves to foreground the role of the environment and other social factors in biosocial processes [7, 49–51]. There is reason to be optimistic that concepts and tools from both the biological and social sciences can be productively integrated if we proceed with caution. First and foremost, it is important to avoid approaches that lead to victim-blaming. Sure, individuals differ in genetic endowments in ways that influence developmental outcomes, both good and bad. And yes, individuals make decisions, good and bad, that matter to their well-being. But none of this happens in a vacuum. Genes only matter if they are expressed, and expression is influenced by the environments—past and present—an individual inhabits. Social structures and hierarchies provide opportunities to those with advantage, but also constrain autonomy and options available to those who are disadvantaged by virtue of their social position.

Second, we should aim to form research teams that are diverse, both in terms of disciplinary background and axes of social identity. We should seek to engage in conversation and collaboration with members of the communities we study. With a wider range of backgrounds and perspectives informing our research, we are more likely to pose novel research questions and less likely to produce results that are misinterpreted or misappropriated.

And third, in formulating our research questions and disseminating our findings we need to be mindful of the cultural and political contexts within which they will be consumed. For example, individuals born at the lower end of the birth weight distribution are at increased risk for cardiometabolic disease later in life [52]. Scientists working in this area understand that the long term health effects of gestational environments are probabilistic, relatively small in magnitude, and shaped by multiple factors over the course of development. Among the general public, however, there is a long history of scapegoating mothers for poor developmental outcomes, and research emphasizing the prenatal environment has the potential to impose even greater burdens of responsibility on pregnant women [53]. We can anticipate this response and communicate our results in ways that underscore the complexity and contingency of development, and we can frame research questions that move beyond an exclusive focus on pregnant women to consider, for example, the role fathers play in shaping the health of their children, or the impact of social and structural factors on shaping risk for lower birth weight deliveries.

## COVID-19 as case study

Human biology is a social biology, and the global pandemic underscores this point at multiple levels. SARS-CoV-2—the virus that causes the clinically defined disease COVID-19—binds to a cellular receptor (angiotensin-converting enzyme 2, ACE2) that is widely expressed in our airways. The virus then gains entry into our cells, hijacks intracellular machinery to generate more copies of itself, and then releases virions to infect more cells. Some of these virions are exhaled as airborne particles and respiratory droplets. At this point, how severe an infection will be, and whether it can be spread to other people, largely reflects the outcome of a race between virion production and expansion, and the ability of our immune system to contain and eliminate the virus.

SARS-CoV-2 infection can therefore be understood as largely a product of processes inside the body, and this has been the primary focus of clinical, virological, and immunological research on the pandemic. However, as the scientific community and general population has increasingly become aware, SARS-CoV-2 infection can also be understood as a product of outside the body processes involving social interactions and behavior in group settings. Infections are only possible for the exposed, the virus spreads through close social contact, and the likelihood of exposure is only partially under an individual’s control. Isolation, mask-wearing, and vaccination are very effective at limiting viral transmission in the community, but only if enacted by substantial numbers of people. Risk of infection is therefore an emergent property of social networks (and the attitudes and behaviors of people in them), of local norms and policies, and the broader cultural and political landscapes that condition the likelihood that individuals will adopt preventive behaviors in particular communities. Furthermore, risk is not evenly distributed within communities: crowded living conditions, reliance on public transportation, and employee designation as an “essential worker” all contributed to higher exposure risk early in the pandemic and helped drive inequities in COVID-19 morbidity and mortality [54, 55]. The social world clearly shapes the likelihood of infection, but the converse is also true: when COVID-19 case rates increase in a local community, residents are more likely to adopt protective behaviors and to support public policies aimed at reducing community spread [56]. Bidirectional associations between social behavior and infection risk are characteristic of all waves of the pandemic, including those fueled by emerging variants (which are themselves a product of these associations).

The dynamic between processes inside and outside the body plays out in other arenas as well. High burdens of psychosocial stress impair multiple aspects of immune defenses, and likely influence whether an infection is mild or asymptomatic, or whether it leads to hospitalization or death. Social isolation—a direct consequence of mitigation measures like quarantine, travel restrictions, and remote work/school arrangements—is a well-established risk factor for morbidity and mortality, and may extend the health impacts of the pandemic beyond COVID-19 [57, 58]. Much attention has been given to “pre-existing” conditions that can increase the severity of COVID-19; less attention has been given to the fact that these conditions are not equitably distributed in the US. For example, diabetes, obesity, and cardiovascular disease—all of which increase the risk of severe COVID-19—are more common in communities of color due to histories of discrimination and concentrated disadvantage [54]. Neighborhood maps that highlight COVID-19 “hot spots” in urban environments will identify the areas that are also suffering from high rates of poverty, crime, pollution, and lack of access to fresh foods and open green spaces [55, 59]. In a sense, being a resident in these segregated and disadvantaged communities can be considered a “pre-existing condition” for COVID-19.

The long-term effects of the pandemic on individuals, communities, and societies are not yet known, and biosocial dynamics will play out over the life course, and potentially across generations. For example, rates of gestational diabetes, preeclampsia, and poor fetal growth all increased during 2020 over the prior year [60], which portends a potential cohort effect on future rates of morbidity and mortality, as well as cognitive development and educational attainment among the COVID birth cohort [61]. Among adults, lingering symptoms after infection (described as long COVID, post-COVID-19 syndrome, and/or post-acute sequelae of COVID-19, among other terms) can influence work productivity, physical activity and diet, and interpersonal relationships, all of which shape trajectories of health as we age. For many workers, economic shocks have increased unemployment, reduced wages, and constrained opportunities for upward mobility, particularly for caregivers of small children forced to juggle the demands of work and family during school closures [62]. For children, lost learning due to school closures may reduce educational attainment and widen achievement gaps for those at greatest risk for falling behind [63]. Socioeconomic status is a “fundamental cause” of health, and it will likely play an important role in biosocial loops set in motion by the pandemic for decades to come.

Research into the biology of SARS-CoV-2 infection has been biased toward studies of COVID-19 patients requiring clinical management and hospitalization, or samples enriched with individuals at increased risk of infection due to repeated occupational exposure (e.g., healthcare workers). Yet more than two-thirds of all infections are mild or asymptomatic, and only a small proportion require hospitalization [64]. Clinical studies therefore do not capture the “on the ground” experience of the vast majority of people who get infected with SARS-CoV-2, and have limited utility for tracking viral spread and the development of immunity in the general population. Furthermore, in framing COVID-19 as a clinical phenomenon, we miss an opportunity to illuminate the contextual, interpersonal, and individual factors that explain patterns of exposure and response to infection in the community, where the pandemic is having social, psychological, and economic impacts that go well beyond the clinic and hospital. Moving forward, a biosocial approach can prepare us to respond more effectively to future pandemics, as well as help us understand the lasting impact of this one.

## Conclusion

It has long been fashionable to claim that nature versus nurture is dead. Appeals for more integrative, transdisciplinary approaches to human development and health abound [17, 49]. With an increasingly sophisticated toolkit for measuring biological processes in non-clinical settings, large studies with data that span society to cells and everything in between, and a wide range of analytic and computational methods for making sense of it all, the time is right for a new generation of biosocial research that integrates and advances both the biological and social sciences. The price of integration is vigilance: we cannot deny the potential for biological data to be misused and misinterpreted, and we assume an abiding obligation to challenge simplistic determinism and any attempts to essentialize, stigmatize, or subordinate members of society [65]. Scientists have engaged in biosocial research, in one form or another, for more than 100 years. We now have a golden opportunity to deliver on the promise of a more sophisticated understanding of the inextricable links among society, biology, and health.

## Data Availability

Not applicable.
